# Fixed Forms of Behavior as Excessively Rigid Behavior in Normal and Pathological Individual and Group Systems

**DOI:** 10.11621/pir.2021.0101

**Published:** 2021-03-20

**Authors:** Genrikh V. Zalevskii

**Affiliations:** Immanuel Kant Baltic Federal University, Kaliningrad, Russia

**Keywords:** FFB, fixed forms of behavior, individual and group systems, biopsychosocionoetic model, system-network approach

## Abstract

**Background:**

This article is devoted to the problem of excessively rigid behavior, which the author has named “fixed forms of behavior” (FFB). This term was suggested to me by the concepts of P. Janet (*idée fixe*), S.Freud (*Fixierung*), and D.Uznadze (*fiksirovanaya ustanovka* — fixed set/attitude). By FFB, the author understands a broad spectrum of behaviors of a person or a group of people, which, according to the cultural norms of a given society for persons of a certain age, gender, and status, have become inappropriate, yet are repeated in situations objectively requiring that they change; the degree of realization and acceptance of the need for this change can vary.

**Results:**

Through literature analysis and the collection of experimental data over many years of research, in which over 1,150 persons took part — 550 healthy subjects and 600 mental patients from a broad spectrum — and on the basis of a biopsychosocionoetic model of the nature of man and his health, and a system-network approach, it has become possible to distinguish the following models to explain the nature of fixed forms of behavior: *neurodynamic, energy-economic, phylogenetic, person-environment relationship, dispositional, stressogenic, pathogenic, psychodynamic, learning (behavioral-cognitive), system (an excessively rigid system and structural relations between levels of action).*

## Introduction

By fixed forms of behavior (FFB), I understand a broad spectrum of behaviors of a person or a group of people, which, according to the cultural norms accepted in a given society for persons of a certain age, gender, and status, have become inappropriate, yet are repeated in situations objectively requiring that they change; the degree of realization and acceptance of the need for this change can vary. Such behavior can be defined as inert, sluggish, stagnant, bigoted, rigid, dogmatic, inelastic, non-plastic, inflexible, uncreative, unchangeable, or difficult to change. But the term fixed forms of behavior includes all of those characteristics (Zalevskii, 1976, 1987, 1993, 2003, 2007, 2008, 2013).

The concept of fixed forms of behavior was proposed by me over 40 years ago in the *Zhurnal nevropatologii i psikhiatrii imeni S.S. Korsakova* (Zalevskii & Rogovin, 1970). The term was not in use at that time, but the idea was suggested to me by those of P. Janet (*idée fixe*), S. Freud (*Fixierung*), and D. Uznadze (*fiksirovanaya ustanovka* — fixed set/attitude).

My understanding of fixed forms of behavior has developed considerably since then, but the problem itself is far from having been studied sufficiently.

The best demonstrations of fixed forms of behavior — their nature, variety of manifestations, and place in the lives of people — are situations of social upheaval and rapid transformation (for example, the Perestroika period in the Soviet Union).

The spectrum of fixed forms of behavior is very broad. Throughout our lives there are two types of activity — variable and invariable (or difficult to change), that is, fixed forms of behavior. The relationship between them is one of the key problems of biology and psychology, as well as related sciences: physiology, general and social psychology, personality psychology, and psychopathology.

Fixed forms of behavior can reveal themselves both at the level of the individual personality, that is *individual systems,* as well as at the level of groups of people (families, organizations, societies, and states), i.e., *group systems.* Their influence is observed in different spheres of everyday human activity: in education (in closed educational systems, in difficulties accepting innovative processes), science (unjustified defense of one’s own ideas and “cherished” theories, irrational resistance to ideas offered by colleagues), culture (obsolete customs and traditions, ethnocentrism, fanaticism) (Zalevskii, 1996, 1999, 2008), and in professional activity, for instance, that of psychologists and psychotherapists (“adjusting the client to one’s concept”, adherence to one’s favored methods). Problems of education (re-education) and psychotherapy often involve the need to change fixed forms of behavior (inappropriate habits and stereotypes of behavior, varied forms of inappropriate or deviant behavior (Zalevskii, 1976, 1987, 1999).

I emphasize that, like Janet, I regard behavior (both fixed and non-fixed) as comprising thought, feeling, attitude, and motion. From this standpoint, the opinion of psychotherapist Robert Goulding appears quite justified, that “psychotherapy is a science of how to change oneself — to change one’s thoughts, feelings, behavior, sometimes even one’s body” (Goulding, 1998).

## Methods and Participants

Through theoretical analysis of the specialized literature and data from my own experimental studies over many years (Zalevskii, 1976–2015), in which over 1,150 persons took part — 550 healthy subjects and 600 mental patients from a broad spectrum — and on the basis of a biopsychosocionoetic model of the nature of man and his health, and a system-network approach, it has become possible to distinguish the following models to explain the nature of fixed forms of behavior: ***neurodynamic, energy-economic, phylogenetic, violation of person-environmental relationship, dispositional, stressogenic, pathogenic, psychodynamic, learning (behavioral-cognitive), maladjusted person system (excessive rigidity of the system and violation of the structural relations between levels of action/behavior).***

## Results and Discussion

### 1. The neurodynamic model of fixed forms of behavior

This model explains their nature and causes by the inertness of nervous processes for various reasons — asthenization (fatigue, nervous exhaustion), pathology (as a result of brain damage), etc. But the demonstrated relations between the nervous processes and psychological characteristics of a person reflect only indirect links and low correlations. For instance, in perseveration, one of the fixed forms of behavior, pathological inertness of nervous processes is caused by weakening of attention or of “conscious control of action” (E. Kraepelin).

### 2. The energy-economic model of fixed forms of behavior (habits, routine actions, etc.)

First developed by V.M. Bekhterev (1926), this model is derived from the general principle of energy conservation. There are references to the principle in the works of the Georgian school of the psychology of set (D.N. Uznadze) as well. A mental set corresponding to objective circumstances normally includes some fixed elements gained from past experience, and, in keeping with the principle of energy conservation, guarantees the satisfaction of a need (Prangishvili, 1973, pp. 355–356). But, as is well known, a fixed set, apparently based on the same principle, does not guarantee the satisfaction of a need. Russian scientist A.A. Ukhtomskiy, developing his theory of the dominant, uses examples borrowed from the science of science to demonstrate that the principle of energy conservation, being the essence of the dominant, works well in stereotyped, routine conditions, but produces fixed forms of behavior in conditions that are new and “do not coincide with the dominant”. “I feel it my duty”, wrote Ukhtomskiy, “to point out that many doctrines and theories are biased a priori, because from the outset they are oriented towards stability and minimal action; reality is sacrificed to the beautiful eyes of theory” (Ukhtomskiy, 1973, p. 390).

### 3. The phylogenetic model of fixed forms of behavior

This model is close in many respects to the “energy approach”. Observations by ethologists and experiments of zoopsychologists show that fixed forms of behavior are demonstrated by animals too, and the lower are the organisms on the phylogenetic scale, the more numerous are the manifestations of such forms of behavior. Instinct, directing the behavior of the animal, is adapted to life in a stable environment and functions well in these conditions. But what if the conditions of life change suddenly? Why did the dinosaurs die out so suddenly? It is because instincts are potentially fixed forms of behavior. Convincing illustrations of this can be found in the studies of K. Lorenz, N. Tinbergen, and other ethologists.

### 4. Fixed forms of behavior as a violation of the person-environment relationship

This model goes back to the work of Russian physiologist and psychologist I.M. Sechenov more than a century ago, who wrote: “Always and everywhere life is made by cooperation of two factors — present but changing inner organization and certain influences from outside. It does not matter whether we look at life in terms of its ultimate objective — preservation of the individual — as on something developing, because preservation at each discrete moment of existence is achieved through unceasing transformations. This follows from that fact that in all organisms, the preservation of the whole body and of life itself is achieved not by stability once formed, but by constant partial destruction and rebuilding of the elements of the body” (Sechenov, 1963, pp. 288–289).

From the thoughts of Sechenov, as well as those advanced later by C. Bernard (“on the constancy of internal ambiance”), W. Cannon (“on homoeostasis”), and I. Pavlov (“on balancing”), the conception evolves, first, of harmony between internal conditions and external influences, and, secondly, that this harmony is ensured only when activity and behavior are determined by the constantly changing influences of the ambiance. In the case of fixed forms of behavior, this harmony is broken and behavior is determined only by internal conditions, while disregarding external, objective requirements.

In mental disorders, the determination of the person’s mental activity and behavior by the external world is considerably weakened and even completely disappears. W. Griesinger, one of the founders of scientific psychiatry, wrote that “the essential process of insanity manifests itself predominantly in that certain moods, feelings, emotions, decisions come from within as a consequence of mental illness, while in the normal state our feelings and decisions are caused only by sufficient external motives and therefore manifest a certain link with the external world” (Griesinger, 1881, p. 64). “Stereotypes are actions completely independent from the general situation, which do not correspond to any objective surroundings” (Y. Klasi, cited in Bash, 1955).

### 5. The pathogenic model of fixed forms of behavior

Clinical practice and various clinical studies, including our own, allow us to formulate a pathogenic conception of fixed forms of behavior. It is somewhat global, but can be rendered more concrete by including elements of other schemes, such as psychodynamic and learning (behavioral-cognitive). This is because fixed forms of behavior, first of all, noticeably increase in intensity and extensity from the norm to mental pathology and, secondly, there is a further increase, despite certain qualitative specifics, from mild forms of psychiatric disorders (e.g., neurosis) to more severe forms (e.g., schizophrenia).

These results were obtained with the Tomsk Rigidity Questionnaire developed by me (see Burlachuk, 2008; Zalevskii, 1987; Wasserman & Kudryavtsev, 1985; Pawlik & Rosenzweig, 2000).

#### 5.1. The psychodynamic model of fixed forms of behavior

Within this framework, fixed forms of behavior characteristic of neurosis are interpreted as manifestations of defense mechanisms. “The neurotic protects himself ”, wrote Freud (1948, p. 376), “by shaping the fixed habits leading to the preference of certain ways to solve personal problems”. According to A. Adler (1975), the neurotic tends toward a rigid, maladaptive lifestyle. G. Murphy also considered that “any schematization, any stereotypization, will be a neurotic means of defense” (1947, p. 240).

#### 5.2. The learning (behavioral-cognitive) model of fixed forms of behavior

Advocates of this approach share the opinion that fixed forms of behavior (the tyranny of “should” and irrational thought, inappropriate cognitive schemes and wrong decisions, inappropriate attitudes, bad habits, anxiety and depression, learned helplessness) are the result of “faulty learning”, including social learning, although the specific mechanisms of this learning can be rather different (J. Wolpe, A. Lazarus, A. Bandura, A. Ellis, A. Beck, M. Seligman).

### 6. The maladaptive person model of fixed forms of behavior

The nature of fixed forms of behavior can be explained by the humanistic psychology of Rogers (1961), implemented in person-centered psychotherapy for the “maladaptive person”, as a consequence of “ineffective Ego-concepts” and “incongruence between the Ego-concept and new experience”. Such a condition occurs when a new experience is rejected and distorted, since clients often deny and distort the positive feedback received from the outside, trying to protect their Ego-concept from threats of violation or even from having to replace it with a new one. This mechanism, in terms of growth in the number and intensity of fixed forms of behavior, often synergizes anxiety and tension (intensionality). Tension-related reactions (intensional reactions — in this case FFB) assess an experience with rigid (absolute and hard) positions in the form of excessive generalization, mixing facts, and striving to rely on abstractions without checking their validity.

### 7. The stressogenic model of fixed forms of behavior

Psychologists of different orientations note that the reason for fixed forms of behavior may be connected with alerting, fear, frustration, shock, or stress caused by either powerful and brief or weak and long-acting stressors. The last of these turn out to be particular reasons, under the influence of which the familiar, the accustomed, is so carefully and persistently protected, that hostility is shown to anything new, to changes (Levitov, 1977). Levitov himself, alongside other researchers, considers it possible to think of mental rigidity as a state that emerges as a dispositional factor of fixed forms of behavior. In our studies, we have found experimentally a relationship between the action of stressors and fixed forms of behavior, highlighting this along with trait-rigidity and state-rigidity, manifested separately or with synergistic connections between them. This occurs particularly with nervous psychopathology.

### 8. System models of fixed forms of behavior

#### 8.1. Excessive rigidity of the system of fixed forms of behavior

L. von Bertalanffy (1968) distinguished between open and closed systems: The first can only exchange energy, whereas the second can exchange energy and matter. Interesting in this context is his idea that “a prerequisite for the stability of organic systems is the constant renewal of their elements”. It can be assumed that “closed systems”, not only organic but also social ones, lack this “constant renewal of elements” both within them and in interaction with other systems. This happens according to our concept of “excessive rigidity of the system” (Zalevskii, 2007), because any system striving for stability, by virtue of disruptive feedback, “rushes past” the optimal necessary measure of stability and directs itself toward hyperstability, becoming rigid (stiff , hardened), excessively closed, as evidenced by the increase in the number of fixed forms of behavior and their increased intensity. As a rule, in such cases an individual or group system collapses.

We noted above, albeit in other terms, one of the classics of German psychiatry by Griesinger, on the extreme (pathological) case of closed individual systems. M. Rokeach (1960) wrote about “the open and closed mind”. Could something similar happen with a group system? Probably it could, since this has been characteristic of small groups such as a certain type of family, religious sects, mafia-like formations, and even large social groups such as entire states, for example, the famous totalitarian regimes. We find the philosophical context of this idea already stated by H. Bergson: “The morality of a closed society is static. The morality of an open society is dynamic. …The foundation of open morality is the creative personality; its main characteristic is the spirit of innovation, breaking all the fixed schemas of a closed society” (Bergson, 1932). V. Satir et al. (Satir, Stachowiak, & Taschman, 1975) rightly believed, based on their rich experience of psychotherapeutic work with the family, that in a closed system, people cannot flourish; at best they can only exist, but people need significantly more.

#### 8.2. Violation of the structural relations between levels of behavior

Long-standing experimental studies of personality, both normal and pathological, allow me to offer an original psychological conception of fixed forms of behavior. Its main features are as follows: (a) a hierarchical organization of personality and behavior (activity, actions), in which the personality manifests itself and “personality becomes real” (Hegel); (b) spatial rigidity (embracing the structure and levels of personality), stipulating particular manifestations of fixed forms of behavior; (c) distortion of relationships within the basic structure — between the high level of purpose and the subordinate level of means. Two variants of such distortion are possible: (a) when the fixogenic tendency (a tendency toward FFB) is a means, and (b) when the fixogenic tendency is the purpose of an action (behavior). In the first case, an inadequate means (whether material or ideal) merges with the purpose, making the action maladaptive and leading the person to function on a lower level. In the second case, an inadequate purpose becomes an end in itself, merging with the means, which again makes the action inadequate and leads the person to function on a lower act level. In the third case, both purpose and means can be fixogenic. An example: one purpose and one means — an alcoholic and/or a suicide (hanging oneself) (Durkheim, 1912).

### 9. The dispositional model of fixed forms of behavior

The basis of the dispositional approach to the study of personality is the assumption that “personality is what lies behind specific acts and within the individual” (Allport, 1937) and that a personality trait is a determining or at least defining element of behavior (Allport, 1954). Rigidity was shown to be a core element of all fixed forms of behavior. R. Cattell (1964) even distinguished a particular type: “dispositional rigidity”. A number of studies note that one of the features of “an emotionally sound personality” is flexibility (Rogers, Maslow, Ellis). “Emotionally sound people are intellectually flexible, tend to be open to change,” wrote Ellis, “and are prone to take an undogmatic and unbigoted view of the infinitely varied people, ideas, and things in the world around them. They can be firm and passionate in their thoughts and feelings, but they can also look comfortably at new evidence and often revise their notions of ‘reality’ to conform with this evidence” (Ellis, 1987).

In our studies we also discovered a correlation between the position of the person on the continuum of rigidity-flexibility and features of intensity and extensity of fixed forms of behavior. Furthermore, specific representations of mental rigidity (partial, total, or as a type-forming trait) lead to various “types of rigid personality” (authoritarian, dogmatic, etc.).

## Conclusions

On the basis of the biopsychosocionoetic model of man’s nature and health (Zalevskii & Kuzmina, 2012) and the system-network approach (Guseltseva, 2007), it is become possible to distinguish some models to explain the nature of fixed forms of behavior.

Our list of explanatory models of the nature of fixed forms of behavior is not exhaustive. Quite close to this approach are attempts to explain fixed forms of behavior through other models, for example: *“disruption of the feedback mechanism”* (P.K. Anokhin, A.R. Luria), *“reactions without meaning and value”* (*Leerlaufreaktionen* — K. Bash, K. Lorenz), *“lapse of meaning”* (L. Jakobovits, W. Lambert), *“ignorance of life experience”* (P. Gannushkin, O. Kerbikov), *“non-congruence between I-conception and new experience”* (C. Rogers), *“lazy thinking’*’ (D. Kahneman).

Of course, the explanatory models of fixed forms of behavior described here do not exclude, but rather complement each other.
